# Attributable mortality to healthcare-associated infections: a comprehensive nationwide assessment in Spain, 2022 and 2023

**DOI:** 10.2807/1560-7917.ES.2026.31.7.2500139

**Published:** 2026-02-19

**Authors:** Mireia Cantero, Clara Salamanca, Lina Marcela Parra, Marta Eva González-Pérez, Manuel García de la Vega, Ángel Asensio, on behalf of EPINE Study Group

**Affiliations:** 1 Preventive Medicine Department, Hospital Universitario Puerta de Hierro-Majadahonda, Madrid, Spain; 2 Preventive Medicine Department, Complejo Asistencial Universitario de León, León, Spain; 3 Management Team, Hospital Universitario Juan Ramón Jiménez, Huelva, Spain; 4 Spanish Society of Preventive Medicine, Public Health and Health Management, Madrid, Spain; the members of the study group are acknowledged at the end of the article; * These authors contributed equally to this work and share first authorship.

**Keywords:** attributable mortality, healthcare-associated infections, Spain, point prevalence survey

## Abstract

**BACKGROUND:**

Few studies have quantified mortality caused by healthcare-associated infections (HAIs) of all types.

**AIM:**

This work’s objective was to estimate the overall impact of HAIs on mortality in Spain.

**METHODS:**

Spain performs annually a point prevalence survey of HAIs and antimicrobial use in hospitalised patients. In 2022 and 2023, prospective follow-ups of patients to evaluate their status 30 days after the survey (still admitted, discharged, deceased) were additionally conducted. This information allowed assessing the effect of HAIs on mortality, by logistic regression. We calculated the attributable fraction among the exposed (patients with HAIs) and the population attributable fraction (among all hospitalised patients). Finally, we estimated the annual number of deaths attributable to HAIs.

**RESULTS:**

Of 107,781 inpatients included in the study, 56,323 (52.26%) were males and 51,458 (47.7%) females. Most patients (n = 59,790; 55.47%) were ≥ 65 years old. The HAI prevalence was 7.8% (n = 8,375). Crude mortality rate was 5.7% (5,715/99,406) among patients without HAIs and 11.0% (918/8,375) among those with HAIs. The adjusted odds ratio (AOR) for inpatient mortality associated with HAIs was 1.70 (95%CI: 1.56–1.86). The attributable fraction of deaths due to HAIs among inpatients who died with a HAI was 41.2% and 3.2% among all inpatient deaths. The estimated annual number of inpatient deaths attributable to HAIs in Spain was 6,774.

**CONCLUSION:**

In Spain, HAIs highly impact mortality. The number of deaths attributable to HAIs is over three times that caused by road traffic accidents. Addressing this requires immediate strengthening of infection prevention programmes across healthcare settings and their thorough implementation.

**Key public health message**

**What did you want to address in this study and why?**

Healthcare-associated infections (HAIs) can make patients sicker, prolong their hospital stays and can even lead to deaths. While HAIs are a known problem, few nationwide studies address their overall impact on patients. We aimed to understand the prevalence of HAIs in Spanish hospitals and if HAIs affect patient mortality risk. We also wished to estimate the number of annual deaths attributable to HAIs in Spain.

**What have we learnt from this study?**

We found that about eight of every 100 hospitalised patients in Spanish hospitals had a HAI, and their risk of dying was higher than those without such an infection. We estimate that 6,774 deaths per year in Spain are directly linked to HAIs — more than three times the number of deaths caused by road traffic accidents; hence, HAIs are responsible for a substantial number of deaths in Spain.

**What are the implications of your findings for public health?**

The findings underscore the urgent need to implement effective strategies to reduce HAI rates. Since a considerable proportion of HAIs is preventable, our estimates can help raise awareness among the public, healthcare professionals, and policymakers about the need to establish HAI prevention as a top priority for hospitals and healthcare decisionmakers.

## Introduction

Healthcare-associated infections (HAIs) represent a common and important complication among hospitalised patients. They have considerable socioeconomic consequences, including high mortality, extended length of stay (LOS) and increased costs [1-3].

In Europe, HAIs are estimated to affect 4,500,000 patients and to cause more than 91,130 deaths annually according to reports published in 2016 and 2018 [3,4]. In the United States, research from Klevens et al. estimated the number of deaths caused by HAIs to be 98,987 in 2002 [5]. According to a study from 2011, HAIs increase patients’ LOS and contribute to higher hospital costs, ranging from EUR 1,012 to EUR 19,402 per infection [6].

A high percentage of HAIs (≥ 50%) are considered preventable [6], and various evaluations reported by the World Health Organization (WHO) demonstrate that the incidence of HAIs is effectively reduced by infection prevention and control programmes [7]. Moreover, several studies have shown that hospitals actively implementing such programmes can reduce the risk of HAIs [2,8] and consequently, associated mortality.

A proper assessment of the investment needed for infection prevention and control programmes requires a thorough understanding of the disease burden associated with HAIs. Most studies evaluating the impact of HAIs on mortality have focused on specific wards [9,10], particular types of infections [11] or individual microorganisms [12]. So far, quantifying mortality caused by all types of HAIs has mostly been done through indirect estimates [13,14] or estimates relying on single centre settings [15-17] due to the substantial number of resources needed. Because of this, few multicentre and nationally representative studies have been conducted to date [1,8,18,19].

In this study, we aimed to quantify the impact of HAIs on inpatient mortality in Spanish hospitals using national data.

## Methods

### Study design and setting

The Spanish point prevalence survey (PPS) of HAIs and antimicrobial use in acute care hospitals (EPINE) has been conducted annually for more than 30 years. It is promoted by the Spanish Society of Preventive Medicine, Public Health, and Health Management, and is financed by the Spanish Ministry of Health. Each year, more than 300 hospitals voluntarily participate in the study, collecting data from nearly 60,000 inpatients. This sample accounts for 55% of acute care hospitals and 75% of acute care beds in Spain. Among participating hospitals, 74% have an intensive care unit (ICU).

Since 2012, the Spanish PPS, which constitutes an observational cross-sectional study, has been adapted to align with the European PPS, coordinated by the European Centre for Disease Prevention and Control (ECDC) [20]. In addition to the variables required by the ECDC protocol, the EPINE survey collects supplementary data, such as certain comorbidities and community-acquired infections. Furthermore, since 2022, a prospective follow-up has been implemented for all patients to assess their clinical status 30 days post-survey (i.e. whether they remained hospitalised, were discharged, or had died).

This study analyses data from the EPINE 2022 and 2023 surveys, together with 30-day follow-up data on patient outcomes.

### Data collection

Trained investigators conducted a 1-day survey, completing standardised hospital and patient data collection forms. All inpatients present on the ward before or at 08:00 am on the day of the survey were included. Patient data were collected from patient records and submitted through a web-based data entry system [21].

The following variables were recorded: patient demographics (age; sex as a binary variable: male/female), severity of disease at admission as assessed by the McCabe−Jackson severity score [22], comorbidities (chronic kidney disease (CKD), chronic respiratory disease, liver cirrhosis, coma, diabetes mellitus, hypoalbuminaemia, immunodeficiency, neoplasia, neutropenia and sores), and invasive procedures, including both invasive devices in place on the day of the survey (central venous catheter, intubation, peripheral venous catheter and urinary catheter) and surgery since admission (including the date of surgery). Hospitals were classified by type (primary, secondary, tertiary and specialised), size (< 200, 200–399, 400–649, ≥ 650 beds) and ownership (public and private). Ward specialty was also noted.

Active infections were identified using the ECDC case definitions [20]. A HAI was recorded when signs and symptoms of the infection were present on the survey date, or signs and symptoms had occurred previously and the patient was (still) receiving treatment for that infection on the survey date, and the onset of symptoms was on day 3 post admission or later (with day 1 being the day of admission); or if the patient presented with an infection but had been readmitted less than 48 hours after a previous discharge or transfer from a healthcare facility; or if an invasive device was placed on day 1 or day 2, resulting in a HAI before day 3 [20]. Infections acquired in long-term-care facilities (LTCFs) were excluded to ensure comparability across the 2 years of our study, since HAIs from LTCFs were only included in the Spanish PPS from 2023 onwards. HAIs were categorised into the following groups: respiratory infections, surgical site infections, urinary tract infections, bacteraemia, gastrointestinal infections, and other locations. Respiratory infections were further subdivided into pneumonia, lower respiratory tract infections without pneumonia, and COVID-19. The number of HAIs was also calculated and categorised as either 1 or ≥ 2.

The variables surgery and COVID-19 were recoded as surgery before HAI and COVID-19 before HAI, respectively, based on the dates of surgery and COVID-19 onset in relation to the date of the first HAI. The variable COVID-19 before HAI included both community-acquired infections and HAIs.

The LOS prior to HAI (at the date of the survey) was calculated by subtracting the date of HAI onset from the date of admission. For inpatients without HAI, this variable was determined by subtracting the date of the survey from the date of admission.

Patient outcomes 30 days after the day of the survey were recorded as still admitted, discharged, or dead.

### Statistical analysis

HAI prevalence was calculated as the number of inpatients with at least one HAI per 100 inpatients. We computed HAI prevalence, as well as mortality rates for patients with and without HAIs, stratified by demographic characteristics, McCabe–Jackson severity score, LOS prior to HAI, comorbidities, invasive procedures, hospital features and ward specialty. Patients with missing 30-day follow-up data were excluded from the analysis.

To assess the effect of HAIs on mortality, univariate and multivariate logistic regression analyses were performed. In these analyses, mortality (death) was defined as the dependent variable, while the presence of a HAI was included as an independent variable (Stata 18 software, StataCorp, College Station, US). Both crude (OR) and adjusted odds ratios (AOR) were calculated. The reported OR represents the odds of death among inpatients with a HAI compared with those without HAIs, stratified by the categories of each variable.

We also computed the HAI prevalence and mortality rates according to both the number and type of infections. Patients who presented infections in more than one category were included in the analysis for each relevant category. The OR of death associated with HAIs (both crude and adjusted) was calculated. The reported OR represents the odds of death among inpatients with a specific infection or a given number of infections, compared with those without any HAI.

In the multivariate model, confounding was assessed using the ‘confound’ command in Stata. Potential confounders included sex, age, McCabe score, LOS prior to HAI, surgery before HAI, hospital type, size, ownership, and COVID-19 — whether healthcare or community-acquired — before HAI. A variable was considered a confounder if its inclusion led to a ≥ 10% change in the OR for HAI. This comparison was made across the binary values (0 and 1) of the outcome variable. Variables meeting this threshold were retained in the final model.

Comorbidities, invasive devices, and ICU admission were excluded due to the inability to determine their temporal relationship with the onset of infection, which could introduce reverse causality bias. Although the number of HAIs was initially included in the model, it was subsequently excluded owing to significant collinearity with the HAI variable itself.

In addition, we also explored potential interactions based on theoretical considerations and exploratory analysis. The following interactions were evaluated: sex and age, LOS prior to HAI and surgery, surgery and HAI, hospital size and ownership. Interactions with p values greater than 0.05 were removed.

Model selection was guided by the Akaike information criterion (AIC) and the Bayesian information criterion (BIC), with lower values indicating a better balance between model fit and complexity. A total of 4,045 competing models were compared, and the one with the lowest AIC and BIC values was selected as the best-fitting.

In the final multivariate model, adjustments were made for age, sex, McCabe–Jackson score, surgery before HAI, LOS prior to HAI, hospital ownership, and COVID-19 before HAI (AIC: 36,218.4; BIC: 36,361.4; area under the curve: 0.840).

All significance tests were two-tailed with p values < 0.05 considered statistically significant.

Finally, we calculated:

- The attributable fraction among the exposed (AFe), which indicates the proportion of deaths among inpatients who die with HAIs that can be attributed to HAIs; AFe = (AOR − 1)/AOR.

- The population attributable fraction (PAF), which represents the proportion of deaths in the total inpatient mortality that can be attributed to HAIs; PAF = AFe × HAI prevalence. The PAF was also calculated for both the type and number of HAIs.

The total national number of deaths attributable to HAIs annually was estimated using 2018 inpatient mortality data published by the Spanish National Institute of Statistics (INE) [23], along with the inpatient PAF; total deaths due to HAIs = inpatient deaths in Spain (INE) × PAF.

## Results

### Patients’ demographic characteristics and prevalence of HAIs in Spain

The 2022 and 2023 PPS included a total of 115,185 inpatients, of whom 107,781 (52,002 in 2022 and 55,779 in 2023) had follow-up data available, representing 93.6% of the total. Among the 107,781 patients included in the study, 52.3% of the patients were males and more than half of them were 65 years old or older (55.5%) (Table 1). The patients’ demographics as well as the severity of disease (McCabe–Jackson score) are summarised in Table 1. The prevalence of HAIs in Spain during 2022 and 2023 was estimated to be 7.8% (n = 8,375).

**Table 1:** Estimates of HAI prevalence, inpatient mortality and the effect of HAIs on mortality risk, by patient demographic characteristics, Spain, 2022–2023 (n = 107,781 patients)

Patient characteristics	Patients			Prevalence of HAIs		Mortality in patients with HAIs		Mortality in patients without HAIs		Crude estimator		Adjusted estimator		p value
	Number	Total	%	Number	%^a^	Number	%^b^	Number	%^c^	OR	95%CI	AOR^d^	95%CI	
**Sex**														
Female	51,458	107,781	47.7	3,541	6.9	390	11.0	2,509	5.2	2.24	2.00–2.51	1.85	1.62–2.11	< 0.001
Male	56,323	107,781	52.3	4,834	8.6	528	10.9	3,206	6.2	1.85	1.68–2.04	1.60	1.43–1.80	< 0.001
**Age group**														
< 16 years old	7,993	107,781	7.4	331	4.1	18	5.4	52	0.7	8.42	4.87–14.55	4.77	2.35–9.68	< 0.001
16–64 years old	39,995	107,781	37.1	2,802	7.0	204	7.3	1,03	2.8	2.76	2.36–3.22	1.76	1.46–2.11	< 0.001
≥ 65 years old	59,790	107,781	55.5	5,242	8.8	696	13.3	4,633	8.5	1.65	1.51–1.80	1.79	1.63–1.96	< 0.001
**McCabe**														
Non-fatal	82,500	107,781	76.5	5,432	6.6	293	5.4	1,81	2.3	2.37	2.09–2.69	2.18	1.90–2.49	< 0.001
Ultimately fatal	19,304	107,781	17.9	2,174	11.3	285	13.1	1,615	9.4	1.45	1.27–1.66	1.57	1.36–1.81	< 0.001
Rapidly fatal	5,977	107,781	5.6	769	12.9	340	44.2	2,29	44.0	1.01	0.87–1.18	1.21	1.03–1.42	< 0.022
**Length of stay prior to HAI**														
1–3 days	46,261	107,781	42.9	2,919	6.3	235	8.1	1,633	3.8	2.24	1.94–2.58	1.77	1.50–2.10	< 0.001
4–7 days	25,042	107,781	23.2	1,281	5.1	147	11.5	1,416	6.0	2.05	1.71–2.45	1.96	1.59–2.42	< 0.001
8–14 days	17,316	107,781	16.1	1,482	8.6	186	12.6	1,283	8.1	1.63	1.38–1.92	1.62	1.34–1.96	< 0.001
15–29 days	10,929	107,781	10.1	1,356	12.4	189	13.9	938	9.8	1.49	1.26–1.76	1.51	1.24–1.83	< 0.001
≥ 30 days	8,233	107,781	7.6	1,337	16.3	161	12.0	445	6.5	1.98	1.64–2.40	1.60	1.29–1.98	< 0.001
**TOTAL**	**107,781**	**100**	**100**	**8,375**	**7.8**	**918**	**11.0**	**5,715**	**5.7**	**2.01**	**1.88–2.17**	**1.70**	**1.56–1.86**	**0.000**

AOR:  adjusted odds ratios; CI: confidence interval; HAIs: healthcare-associated infections; OR: odds ratio.

^a^ The percentage‘s denominator is the number of patients with the characteristic in question (i.e. on the same row).

^b^ The percentage’s denominator is the HAI prevalence on the same row.

^c^ The percentage’s denominator (i.e.  number of patients – prevalence of HAI) is derivable from numbers on the same row.

^d^ For the AOR, adjustments were made for age, sex and McCabe–Jackson score, LOS prior to HAI, surgery before HAI, hospital ownership, and COVID-19 before HAI.

### Prevalence of HAIs, mortality rates and effect of HAIs on mortality risk, by patient characteristics

A total of 6,633 inpatients died (6.2%; 6,633/107,781), including 918 patients with HAIs and 5,715 without HAIs. By day 30, crude mortality rates were significantly higher among patients with HAIs (11.0%), than among patients without HAIs (5.7%), with an absolute difference in mortality of 5.3% (95%CI: 4.7–5.8). Patients with HAIs were more than twice as likely to die compared with those without HAIs with an OR of 2.01 (95%CI: 1.88–2.17) (Table 1).

According to sex, HAI prevalence was higher in men than in women (8.6% vs 6.9%, p < 0.001). The mortality rate of patients with HAI was similar for both males and females (10.9% and 11.0%) and the effect of HAIs on mortality was statistically significant in both groups: females (AOR: 1.85; 95%CI: 1.62–2.11) and males (AOR: 1.60; 95%CI: 1.43–1.80).

Prevalence of HAIs increased by age group from 4.1% in the group under 16 years old to 8.8% for the group 65 years and older. Similarly, both mortality in patients with HAIs and in patients without HAIs increased by age group, being the highest for patients over 65 years old (13.3% for infected and 8.5% for non-infected). For all age groups, HAIs increased the risk of mortality, but the risk of mortality associated with HAIs was highest for the youngest group (AOR: 4.77; 95%CI: 2.35–9.68).

Regarding severity of disease of the patients at baseline (McCabe–Jackson score), HAI prevalence increased with severity, and mortality rates also rose with severity in both patients with and without HAIs. The risk of mortality associated with HAIs was inversely related to McCabe score. Crude estimations showed that while HAIs had no effect on mortality in the group of patients with most severe disease (OR: 1.01), they did affect groups with milder disease, with the highest effect for the least severe group of patients (non-fatal disease) (OR: 2.37; 95%CI: 2.09–2.69). On the other hand, AORs were statistically significant across all disease severity groups (Table 1).

The prevalence of HAIs increased with LOS prior to HAI, rising from 6.3% among patients hospitalised for 1–3 days to 16.3% among those admitted for more than 30 days. Mortality rates in patients with HAIs and patients without HAIs increased especially for patients hospitalised for more than 4 days. For all LOS groups, HAIs increased the risk of mortality.

### Prevalence of HAIs, mortality rates and effect of HAIs on mortality risk, by patient comorbidities and invasive procedures

The number of comorbidities in patients is associated with the prevalence of HAIs, ranging from 4.5% in patients without comorbidities to 14.5% in those with three or more comorbidities. Similarly, the mortality rates for both HAI and non-HAI patients increased with the number of comorbidities. Nevertheless, the effect of HAIs on mortality, both crude (OR: 1.24–3.04) and adjusted (AOR: 1.25–2.33), is inversely related to the number of comorbidities (Table 2).

**Table 2:** Estimates of HAI prevalence, inpatient mortality and the effect of HAIs on mortality risk, by patient comorbidities and invasive procedures, Spain, 2022–2023 (n = 107,781 patients)

Patient characteristics	Patients			Prevalence of HAIs		Mortality in patients with HAIs		Mortality in patients without HAIs		Crude estimator		Adjusted estimator		p value
	Number	Denominator	%	Number	%^a^	Number	%^b^	Number	%^c^	OR	95%CI	AOR^d^	95%CI	
**Comorbidities**														
None	44,963	106,553	42.2	2,042	4.5	95	4.7	677	1.6	3.04	2.44–3.79	2.33	1.81–3.01	< 0.001
One factor	30,832	106,553	29.0	2,571	8.3	234	9.1	1,606	5.7	1.66	1.44–1.91	1.80	1.52–2.12	< 0.001
Two factors	19,286	106,553	18.1	1,982	10.3	240	12.1	1,729	10.0	1.24	1.10–1.43	1.25	1.05–1.47	0.010
Three or more	11,475	106,553	10.8	1,664	14.5	334	20.1	1,648	16.8	1.24	1.09–1.42	1.36	1.17–1.59	< 0.001
No comorbidity data	1,228	NA	NA	116	NA	15	NA	55	NA	NA	NA	NA	NA	NA
**Type (patients could have more than one type of comorbidity)**														
CKD	17,129	105,914	16.3	1,845	10.8	351	19.0	1,896	12.4	1.66	1.46–1.88	1.64	1.42–1.90	< 0.001
CRD	15,905	104,968	15.2	1,384	8.7	191	13.8	1277	8.8	1.66	1.41–1.95	1.65	1.37–2.00	< 0.001
Liver cirrhosis	2,475	104,952	2.4	254	10.3	47	18.5	263	11.8	1.69	1.20–2.38	1.69	1.15–2.48	0.007
Coma	1,238	49,957	2.5	221	17.9	62	28.1	312	30.7	0.88	0.64–1.22	0.99	0.66–1.48	0.950
Diabetes mellitus	27,088	105,382	25.7	2,652	9.8	349	13.2	2058	8.4	1.65	1.46–1.86	1.54	1.34–1.78	< 0.001
Hypoalbuminaemia	6,054	48,636	12.5	1,079	17.8	182	16.9	784	15.8	1.10	0.91–1.29	1.33	1.08–1.64	0.008
Immunodeficiency	7,586	105,479	7.2	1,005	13.2	148	14.7	723	11.0	1.40	1.16–1.69	1.41	1.13–1.76	0.002
Neoplasia	23,026	105,082	21.9	2,550	11.1	362	14.2	2,448	12.0	1.22	1.08–1.37	1.32	1.15–1.52	< 0.001
Neutropenia	1,165	50,347	3.3	189	16.2	23	12.2	104	10.7	1.16	0.72–1.88	1.22	0.68–2.18	0.510
Sore	6,194	105,404	5.9	1,087	17.5	217	20.0	903	17.7	1.16	0.98–1.37	1.29	1.06–1.57	< 0.001
No comorbidity or comorbidity type data	19,033	NA	NA	807	NA	29	NA	272	NA	NA	NA	NA	NA	NA
**Invasive procedures**														
None	14,165	107,444	13.2	317	2.2	12	3.8	234	1.7	2.28	1.27–4.13	1.36	0.70–2.66	0.361
One factor	51,491	107,444	47.9	2,813	5.5	268	9.5	2,837	5.8	1.70	1.49–1.94	1.38	1.19–1.61	< 0.001
Two factors	29,776	107,444	27.7	2,994	10.1	305	10.2	1,918	7.2	1.47	1.29–1.67	1.32	1.13–1.54	< 0.001
Three or more	12,012	107,444	11.2	2,245	18.7	333	14.8	708	7.2	2.23	1.94–2.56	1.53	1.30–1.79	< 0.001
No invasive procedure data	337	NA	NA	6	NA	0	NA	252	NA	NA	NA	NA	NA	NA
**Type (patients could have more than one type of invasive procedure)**														
Intubation	2,961	106,608	2.8	894	30.2	204	22.8	375	18.1	1.33	1.10–1.62	1.12	0.90–1.39	0.292
Urinary catheter	22,353	106,847	20.9	3,218	14.4	560	17.4	2,345	12.3	1.51	1.36–1.67	1.36	1.18–1.55	< 0.001
CVC	13,332	106,818	12.5	2,950	22.1	421	14.3	1,14	11.0	1.35	1.20–1.52	1.62	1.46–1.79	< 0.001
PVC	82,055	107,020	76.7	6,114	7.5	664	10.9	4,748	6.3	1.83	1.68–1.99	1.47	1.31–1.66	< 0.001
Surgery	30,049	105,470	28.5	3,439	11.4	247	7.2	531	2.0	3.80	3.25–4.44	2.12	1.78–2.52	< 0.001
No invasive procedure or invasive procedure type data	13,443	NA	NA	289	NA	10	NA	206	NA	NA	NA	NA	NA	NA

AOR:  adjusted odds ratios; CI: confidence interval; CKD: chronic kidney disease; CRD: chronic respiratory disease; CVC: central vascular catheter; HAI: healthcare-associated infection; NA: not applicable; OR: odds ratio; PVC: peripheral vascular catheter.

^a^ The percentage‘s denominator is the number of patients with the characteristic in question (i.e. on the same row).

^b^ The percentage’s denominator is the HAI prevalence on the same row.

^c^ The percentage’s denominator (i.e.  number of patients – prevalence of HAI) is derivable from numbers on the same row.

^d^ For the AOR, adjustments were made for age, sex and McCabe–Jackson score, LOS prior to HAI, surgery before HAI, hospital ownership, and COVID-19 before HAI.

The most common comorbidities presented by patients were diabetes mellitus (25.7%), neoplasia (21.9%), and CKD (16.3%). The highest prevalence of HAIs was in patients presenting with coma (17.9%), hypoalbuminaemia (17.8%), and sores (17.5%). However, the most pronounced effect of HAIs on mortality, both crude and adjusted, was found in patients with liver cirrhosis (OR: 1.69; AOR: 1.69), chronic respiratory disease (OR: 1.66; AOR: 1.65) and CKD (OR: 1.66; AOR: 1.64).

The number of invasive procedures presented by the patients was associated with the prevalence of HAIs and ranged from 2.2% for patients with non-invasive procedures to 18.7% for patients with three or more invasive procedures. Likewise, the mortality rates for both HAI and non-HAI patients increased with the number of invasive procedures. The adjusted effect of HAIs on mortality is higher among patients who have undergone three or more invasive procedures (AOR: 1.53) (Table 2).

The most common invasive procedures were peripheral venous catheter (76.7%) and surgery (28.5%). The prevalence of HAIs was highest for patients with intubation (30.2%), followed by patients with central venous catheter (22.1%). The highest impact of HAIs on mortality, both crude and adjusted, was seen in patients undergoing surgery (OR: 3.80; AOR: 2.12).

### Prevalence of HAIs, inpatient mortality and the effect of HAIs on mortality risk, by hospital characteristics and ward specialty

The majority of patients (45.3%) were hospitalised in the largest hospitals (≥ 650 beds), which also appeared to have the highest prevalence of HAIs (8.8%). However, the highest mortality rates in patients with HAIs (12.7–12.9%) and the highest adjusted effect of HAIs on mortality were observed in smaller hospitals (AOR: 1.91–2.17) (Table 3).

**Table 3:** Estimates of HAI prevalence, inpatient mortality and the effect of HAIs on mortality risk, by hospital characteristics and ward specialty, Spain, 2022–2023 (n = 107,781 patients)

Hospital characteristics	Patients			Prevalence of HAIs		Mortality in patients with HAIs		Mortality in patients without HAIs		Crude estimator		Adjusted estimator		p value
	Number	Denominator	%	Number	%^a^	Number	%^b^	Number	%^c^	OR	95%CI	AOR^d^	95%CI	
**Hospital size**														
< 200 beds	18,134	107,781	16.8	1,010	5.6	128	12.7	1,032	6.0	2.26	1.86–2.75	2.17	1.72–2.73	< 0.001
200–399 beds	22,306	107,781	20.7	1,607	7.2	207	12.9	1,248	6.0	2.30	1.97–2.70	1.91	1.59–2.32	< 0.001
400–649 beds	15,209	107,781	14.1	1,194	7.9	130	10.9	872	6.2	1.84	1.52–2.24	1.60	1.28–1.99	< 0.001
≥ 650 beds	48,819	107,781	45.3	4,294	8.8	428	10.0	2,382	5.3	1.96	1.76–2.18	1.58	1.39–1.79	< 0.001
Missing data	3,313	NA	NA	270	NA	25	NA	181	NA	NA	NA	NA	NA	NA
**Hospital type**														
Primary	9,356	107,781	8.7	666	7.1	76	11.4	621	7.1	1.67	1.30–2.15	1.61	1.20–2.15	< 0.001
Secondary	33,566	107,781	31.1	2,459	7.3	298	12.1	2,039	6.6	1.97	1.73–2.24	1.58	1.35–1.84	< 0.001
Tertiary	61,087	107,781	56.7	4,924	8.1	517	10.5	2,873	5.1	2.18	1.97–2.40	1.79	1.59–2.00	< 0.001
Specialised	705	107,781	0.7	71	10.1	4	5.6	8	1.3	4.67	1.37–15.93	4.83	1.14–20.43	0.032
Missing data	3,067	NA	NA	255	NA	23	NA	174	NA	NA	NA	NA	NA	NA
**Ownership**														
Public	91,148	107,781	84.6	7,302	8.0	792	10.8	5,033	6.0	1.91	1.76–2.06	1.63	1.48–1.78	< 0.001
Private	12,991	107,781	12.1	776	6.0	96	12.4	484	4.0	3.42	2.71–4.32	2.69	2.03–3.56	< 0.001
Missing data	3,642	NA	NA	297	NA	30	NA	198	NA	NA	NA	NA	NA	NA
**Ward specialty**														
Surgical specialty	22,741	107,781	21.1	2,223	9.8	111	5.0	514	2.5	2.05	1.67–2.52	1.84	1.44–2.34	< 0.001
Chronic	336	107,781	0.3	59	17.6	11	18.6	26	9.4	2.21	1.02–4.78	3.26	1,24–8.59	0.017
Geriatric	1,587	107,781	1.5	140	8.8	29	20.7	226	15.6	1.41	0.92–2.18	1.73	1.03–2.89	0.037
Medical specialty	40,063	107,781	37.2	2,945	7.4	420	14.3	3,367	9.1	1.67	1.49–1.86	1.45	1.28–1.65	< 0.001
Mixed	23,061	107,781	21.4	1,441	6.2	103	7.1	1,026	4.7	1.55	1.25–1.91	1.35	1.06–1.73	0.015
Neonatology	1,610	107,781	1.5	85	5.3	3	3.5	16	1.0	3.45	0.99–12.08	6.55	1.69–25.39	0.007
Obstetrics/gyneacology	5,549	107,781	5.2	89	1.6	4	4.5	27	0.5	9.47	3.24–27.65	3.79	1.04–13.80	0.042
Other	102	107,781	0.1	8	7.8	3	37.5	3	3.2	18.20	2.9–114.16	5.02	0.05–545.18	0.499
Paediatrics	2,645	107,781	2.5	115	4.3	8	7.0	6	0.2	31.45	10.72–92.25	8.72	2.26–33.59	0.002
Psychiatry	4,340	107,781	4.0	73	1.7	0	0.0	8	0.2	1.00	NA	NA	NA	NA
Rehabilitation	544	107,781	0.5	54	9.9	2	3.7	10	2.0	1.85	0.39–8.65	1.30	0.16–10.27	0.803
Intensive care unit	5,203	107,781	4.8	1,143	22.0	224	19.6	486	12.0	1.79	1.51–2.13	1.33	1.09–1.62	0.005
Missing data	0	NA	NA	0	NA	0	NA	0	NA	NA	NA	NA	NA	NA

AOR:  adjusted odds ratios; CI: confidence interval; HAI: healthcare-associated infection; NA: not applicable; OR: odds ratio.

^a^ The percentage‘s denominator is the number of patients with the characteristic in question (i.e. on the same row).

^b^ The percentage’s denominator is the HAI prevalence on the same row.

^c^ The percentage’s denominator (i.e.  number of patients – prevalence of HAI) is derivable from numbers on the same row.

^d^ For the AOR, adjustments were made for age, sex and McCabe–Jackson score, LOS prior to HAI, surgery before HAI, hospital ownership, and COVID-19 before HAI.

By hospital type, the largest proportion of treated inpatients was in tertiary care hospitals (56.7%), with HAI prevalence also being higher in such hospitals, as well as in specialised hospitals (8.1% and 10.1%). Moreover, while mortality in patients with HAIs was higher in primary and secondary care hospitals (11.4% and 12.1%), the greatest adjusted impact of HAIs on mortality was identified among patients admitted to specialised and tertiary care hospitals (AOR: 4.83 and 1.79, respectively).

Public hospitals served the greatest share of patients (84.6%), and showed the highest prevalence of HAIs (8.0%); nevertheless, the adjusted effect of HAI on mortality was higher for patients admitted to a private hospital (AOR: 2.69).

Most patients were located on medical (37.2%), surgical (21.1%) and mixed wards (21.4%). Prevalence of HAIs was higher in ICUs (22.0%), chronic (17.6%), surgical (9.8%) and geriatric wards (8.8%). Similarly, the mortality rates for both HAI and non-HAI patients were higher in intensive care, chronic, surgical and geriatric wards. However, the greatest impact of HAIs on mortality, was observed in patients admitted in paediatric (AOR: 8.72) and neonatology wards (AOR: 6.55) (Table 3).

### Prevalence of HAIs, inpatient mortality and the effect of HAIs on mortality risk, by number and type of HAIs

A total of 891 patients had two or more HAIs, corresponding to a prevalence of 0.8%. Mortality was higher among patients with two or more HAIs (16.4%) compared with those with a single HAI (10.3%). The AORs for mortality associated with HAIs, compared with patients without any HAIs, were 1.75 for those with one HAI and 1.87 for those with two or more HAIs (Table 4).

**Table 4:** Estimates of HAI prevalence, inpatient mortality and the effect of HAI on mortality risk, by number and type of HAIs, Spain, 2022–2023 (n = 107,781 patients)

HAIs	HAIs			Deaths		Crude estimator		Adjusted estimator			PAF
**Number**	**Number**	**Relative frequency ** **(%)^a^**	**Prevalence ** **(%)^b^**	**Number**	**Mortality ** **(%)^c^**	**OR**	**95%CI**	**AOR** ^d^	**95%CI**	**p value**	**PAF**
**1**	**7,484**	**89.4**	**6.9**	**772**	**10.3**	**1.88**	**1.74–2.09**	**1.75**	**1.47–2.08**	** < 0.001**	**2.96**
** ≥ 2**	**891**	**10.6**	**0.8**	**146**	**16.4**	**3.21**	**2.68–3.84**	**1.87**	**1.21–2.99**	**0.005**	**0.37**
**Type**	**Number**	**Relative frequency ** **(%)^e^**	**Prevalence ** **(%)^b^**	**Number**	**Mortality ** **(%)^c^**	**OR**	**95%CI**	**AOR** ^d^	**95%CI**	**p value**	**PAF**
**Respiratory**	**2,414**	**26.1**	**2.2**	**405**	**16.8**	**3.21**	**2.87–3.58**	**2.29**	**2.01–2.61**	** < 0.001**	**1.24**
Pneumonia	1,171	12.6	1.1	240	20.5	4.03	3.50–4.66	2.92	2.46–3.48	< 0.001	0.71
LRTI without pneumonia	500	5.4	0.5	82	16.4	3.01	2.38–3.82	2.44	1.85–3.22	< 0.001	0.27
COVID-19	799	8.6	0.7	96	12.0	2.10	1.69–2.60	1.39	1.08–1.78	0.009	0.21
**Surgical site**	**2,028**	**21.8**	**1.9**	**84**	**4.1**	**0.65**	**0.53–0.82**	**1.15**	**0.90–1.46**	**0.268**	**0.25**
**Urinary**	**1,631**	**17.5**	**1.5**	**189**	**11.6**	**2.03**	**1.74–2.36**	**1.32**	**1.10–1.58**	**0.003**	**0.37**
**Bacteraemia**	**1,262**	**13.5**	**1.2**	**167**	**13.2**	**2.36**	**2.00–2.78**	**1.68**	**1.38–2.03**	** < 0.001**	**0.47**
**Gastrointestinal**	**745**	**8.0**	**0.7**	**84**	**11.3**	**1.95**	**1.55–2.45**	**1.63**	**1.25–2.12**	** < 0.001**	**0.27**
**Other locations**	**1,187**	**12.7**	**1.1**	**139**	**11.7**	**2.04**	**1.71–2.44**	**1.63**	**1.32–2.02**	** < 0.001**	**0.43**

AOR:  adjusted odds ratios; CI: confidence interval; HAIs: healthcare-associated infections; LRTI: lower respiratory tract infections; OR: odds ratio; PAF: population attributable fraction.

^a^ The percentage‘s denominator is the number of patients with HAIs (n = 8,375).

^b^ The percentage’s denominator is the HAI prevalence on the total number of patients included in the study (n = 107,781).

^c^ The percentage’s denominator is the numbers of patients with HAIs on the same row.

^d^ For the AOR, adjustments were made for age, sex and McCabe–Jackson score, LOS prior to surgery, surgery before HAI, hospital ownership, and COVID-19 before HAI.

^e^ The percentage‘s denominator is the number of HAIs (n = 9,267).

Regarding the type of HAIs, the most frequent were respiratory infections, surgical site infections and urinary tract infections (26.0%, 21.9% and 17.6%, respectively). Among the respiratory infections, nearly one third were associated with severe acute respiratory coronavirus 2 (SARS-CoV-2) infection. Crude mortality was highest for respiratory (16.8%), bacteraemia (13.2%), and urinary tract infections (11.6%). Except for surgical site infections, all types of infections showed a statistically significant association between HAI and mortality after adjustment compared with patients without any HAIs. The highest effect of HAI on mortality, both crude and adjusted, were for pneumonias (OR: 4.03; AOR: 2.92), lower respiratory tract infections without pneumonia (OR: 3.01; AOR: 2.44) and bacteraemias (OR: 2.36; AOR: 1.68). Among respiratory infections, COVID-19 had the lowest impact on mortality (AOR: 1.39) though statistically significant and similar to urinary tract infections (AOR: 1.32) (Table 4).

### Impact of HAIs on mortality

Adjusting for age, sex and McCabe-Jackson score (baseline status), LOS prior to HAI, surgery before HAI, hospital ownership, and COVID-19 before HAI, the effect of HAIs on mortality for the whole cohort OR was 1.70 (95%CI: 1.56–1.86) (Table 5).

**Table 5:** Adjusted odds ratios for mortality Spain, 2022–2023 (n = 107,781 patients)

Associated factors		Adjusted model		
		OR	95%CI	p
HAI		1.70	1.56–1.86	0.000
Male		1.07	1.01–1.14	0.015
Age group	16–64 years old	2.40	1.95–3.21	0.000
	≥ 65 years old	5.78	4.53–7.39	0.000
McCabe	Ultimately fatal	3.02	2.83–3.24	0.000
	Rapidly fatal	22.25	20.72–23.89	0.000
Length of stay prior to HAI	4–7 days	1.20	1.11–1.30	0.000
	8–14 days	1.51	1.39–1.64	0.000
	15–29 days	1.90	1.74–2.08	0.000
	≥ 30 days	1.49	1.34–1.68	0.000
Surgery (before HAI)		0.45	0.41–0.49	0.000
Private ownership		0.76	0.69–0.84	0.000
COVID-19 (before HAI)		1.55	1.38–1.73	0.000

CI: confidence interval; HAI: healthcare-associated infection; OR: odds ratio.

The attributable fraction of deaths due to HAIs among inpatients who died with a HAI was 41.2% (Table 6), and 3.2% among all inpatient deaths. The population-attributable mortality was higher for respiratory infections and bacteraemias (1.24 and 0.47, respectively).

**Table 6:** National estimates of HAIs impact on inpatient mortality per year in Spain

National estimates	Estimator	95%CI
HAI prevalence (%)	7.8	7.6–7.9
AOR HAI mortality	1.70	1.56–1.86
Attributable fraction of deaths due to HAIs among inpatients who died with a HAI (AFe, %)	41.2	35.9–46.2
Population attributable fraction of deaths due to HAIs among all inpatient deaths (PAF, %)	3.2	2.7–3.7
Patient admissions (INE) (number)	4,899,954	
Inpatient mortality (INE) (%)	4.36	
Deaths attributable to HAIs (number)	6,774	5,768–7,904

AFe: attributable fraction among the exposed; AOR:  adjusted odds ratios; HAI: healthcare-associated infection; INE: National Institute of Statistics; OR: odds ratio; PAF: population attributable fraction.

For an expected number of hospital discharges in Spain of 4,899,954 and a hospital mortality of 4.36%, as reported by the Spanish National Institute of Statistics [23]; and a PAF of 3.2%, we estimated an annual number of deaths attributable to HAIs in Spain of 6,774 (Table 6).

## Discussion

In this work, we explored the prevalence of HAIs and the mortality rates in Spanish hospitals in patients with and without HAIs, to assess the risks that such infections pose and their impact on mortality in Spain. Putting our results into a broader context is challenging not only because there are few reports of investigations similar to ours, but also because study designs vary, with for example cohort studies, case−control studies and prevalence-based cohort studies. Comparing findings is further complicated by the differing statistical methods employed, such as the use of hazard ratios, absolute mortality risk differences, attributable fraction among exposed, PAF or indirect estimates.

In terms of HAI prevalence, data from EPINE published in the last 10 years, indicated a decrease over time in Spain [21], reaching 7.8% in years 2022–2023 as reported here. On the other hand, the European 2022–2023 PPS of HAIs and antimicrobial use, estimated the European Union/European Economic Area HAI prevalence to be 7.1%, with that in Spain in the 75^th^ percentile, at 8.2% [24]. Contrasting the European PPS result to ours, should however consider that unlike the 2022–2023 European PPS, our analysis excluded infections acquired in LTCFs – which were only recently classified as HAIs in the European survey [20], and considered as such in EPINE data starting 2023. Removing infections acquired in LTCFs in our study, ensured comparability of data across its 2 years (i.e. 2022 and 2023). 

Concerning the crude mortality rate (unadjusted rate) for patients with HAIs we found a value of 11.0%, which is analogous to those in other European countries such, as Belgium (12.8%; 2007) [18] and Greece (9.7%; 2012) [8]. Overall, in our research, the AOR for inpatient mortality associated with HAIs was 1.70 (95%CI: 1.56–1.86), but we also explored risk of HAI-related mortality in patients, across their characteristics (e.g. comorbidities, procedures that they had undergone), as well as ward specialty or hospital features (e.g. private/public hospital) and numbers/types of HAIs. Like in a Chinese study, comorbidities such as diabetes mellitus (22.4 vs 25.7% in our research) and neoplasia (17.5 vs 21.9%) were common in our patients [25]. 

While as described in other studies [15,17,26], the mortality rates were higher in patients who had comorbidities at time of admission, we observed that the group of patients without comorbidities had a higher probability of dying of a HAI than the group with > 3 comorbidities (AOR: 2.33 vs 1.36). This suggests that in the population without comorbidities, HAIs have a greater impact on mortality, whereas in the population with pre-existing conditions, comorbidities have a major contribution to the fatal outcome. Considering the most common invasive procedures, surgery was the risk factor that had the greatest positive association with the increase of mortality risk of HAI (AOR: 2.12) in our study.

Intensive care and geriatric wards had the highest HAI-related mortality rates (19.6%, 20.7%, respectively). In the literature, ICU has been previously described as a setting with high HAI-related mortality rates [10,26]. However, the probability of death associated with HAIs in the ICU decreased when adjusting for patient’s baseline characteristics. A study carried out in Brazil had similar findings [26], suggesting that these may reflect the greater baseline severity of the condition of patients admitted to ICU. Thus, like for the number of pre-existing comorbidities, the greater severity of illness of patients at ICU admission can reduce the relative contribution of HAIs to the risk of death. It should also be noted that patients in ICU are usually exposed to a greater number of invasive procedures [26].

As regard to hospital ownership, our study described its influence on the risk of dying. In private hospitals, there was a higher probability of mortality due to HAIs compared with public hospitals (AOR: 2.69 vs 1.63). It would be interesting to compare the characteristics of the HAIs reported in both types of hospitals since the HAI prevalence in private hospitals is lower than in public hospitals.

With respect to the main type of HAIs, the most frequent in our country were respiratory followed by surgical site and urinary tract infections (26.0%, 21.9% and 17.6%, respectively). Respiratory infections also predominated in the most recent PPS published by the ECDC but with a reversed prevalence order of surgical site infection and urinary tract infections compared with our findings, possibly due to inclusion in this survey of HAIs acquired in LTCF [24]. In our study, respiratory infections and bacteraemia (AOR: 2.29 and 1.68, respectively) had the highest effect on mortality risk, as reported in other European countries such as Greece in 2012 (AOR: 1.7 and 2.0) [8], Norway in 2004–2011 (AOR: 1.9 and 2.7) [17], or in Belgium in 2007 (AOR: 2.19 and 1.73) [18]. Although the impact of healthcare-associated SARS-CoV-2 infection was lower than that observed for pneumonia, it nonetheless highlights the importance of preventing such infections — typically associated with community transmission — within the hospital setting.

In non-adjusted analyses, surgical site infection was found to have a negative effect on mortality (OR < 1). Because surgical site infections included superficial, deep, and organ-space infections, a sub-analysis was done (data not shown), and this revealed that mortality was lower for superficial infections compared with deep infections and organ-space infections, yet all ORs were below 1. Adjusted estimates, on the other hand, show surgical site infections have no significant effect on mortality. This finding aligns with results reported in other studies [8,17,27].

The multivariable logistic regression model enabled us to estimate the independent effect of HAIs on inpatient mortality while adjusting for key confounders, including age, sex, baseline disease severity (McCabe score), LOS prior to HAI, surgery before HAI, hospital ownership, and COVID-19 before HAI. The inclusion of COVID-19 considered the temporal context of our study (2022–2023), during which SARS-CoV-2 continued to circulate and impact hospitalised patients. Notably, COVID-19 before HAI was associated with an increased risk of death (AOR: 1.55), supporting its role as an independent predictor of mortality. Other variables such as comorbidities, invasive procedures, or ICU admission were not included in the final model due to the inability to establish a clear temporal relationship with the onset of HAIs. Including such variables could have introduced reverse causality bias, potentially distorting the effect estimates of HAIs on mortality.

Regarding the impact of HAIs, in Spain, we found that the attributable fraction of deaths due to HAIs among inpatients who die with a HAI was 41.2% in 2022–2023. This was higher than the 31.9% reported in Finland in 2005 [1] and the 21.3% observed in a single-centre study conducted in our country in 1990 [16]. The PAF in our research (3.2%), which represented the proportion of in-hospital deaths that would not have occurred had the HAIs been absent, is in line with findings of Fabbro-Peray et al. in a French hospital in 2001−2003. These authors report an adjusted population-attributable fraction of 1.7% (95CI%: 1.4−2.1) when adjusting for the McCabe score and 3.0% (95%CI: 2.5−3.4) when adjusting for the Charlson index [15].

The PAF is a key indicator for assessing public health threats, as this value considers not only the effect of a risk factor on mortality but also its prevalence within the target population. Given its relevance for public health decision-making, Von Cube et al. emphasise the importance of accurately quantifying and reporting attributable mortality [28]. They emphasise that the population attributable burden should be linked to timely allocation of financial and human resources. Supplementary Table S1 summarises the main findings on the national-level impact of HAIs in various countries in recent years. Except for Fabbro-Peray’s study [15], we did not identify any other investigation reporting the PAF for HAIs.

Our current work moreover estimated that 6,774 deaths annually are attributable to HAIs in Spain. Using the methodology developed by the Burden of Communicable Diseases in Europe (BCoDE) project to assess the burden of HAIs in individual countries, a study based on 2011 PPS data from Germany estimated that HAIs accounted for 16,245 annual attributable deaths in this country [13]. Taking into account differences in population size, this would suggest that Spain has a proportionally lower number of deaths attributable to HAIs, despite having a higher prevalence of HAIs. The result of the study in Germany is not fully comparable to our finding for Spain, due to distinct methodologies used, whereby the number of attributable deaths in Germany was estimated based on a model published by the ECDC PPS [29], while in our study, deaths were directly observed among the inpatients included in our survey and attributable mortality was calculated using adjusted estimates. 

Nevertheless, HAIs constitute the most important complication for inpatients in Spain and while not being an official cause of death, the number of deaths they resulted in was comparable to other relevant causes, such as road traffic accidents (1,750) or breast cancer (6,492), according to the 2023 mortality data from the Spanish National Institute of Statistics [30]. Given that 50% of HAIs are considered preventable [6], it is reasonable to expect that, with full implementation and compliance with infection prevention and control measures, up to half of HAI-related deaths could be avoided.

This study’s limitations include those linked to the cross-sectional design, in which inpatients with more severe diseases and with a longer hospital stay could be overrepresented. Comorbidities and invasive devices were not included in the final multivariate adjustment as we did not know whether they were present before the HAI or not. Nonetheless, the introduction into the model of severity of disease (McCabe) could counteract the effect of not including the comorbidities [21]. Secondly, a sample bias may have occurred as 6.4% of patients had missing follow-up data. In any case, given that the number of excluded patients was less than 10% and considering the large sample size, we do not assume that this can lead to a systematic bias.

Moreover, the voluntary nature of hospital participation in the EPINE survey could represent an important limitation. Although the participation of hospitals is not random, the large size of the study population, which represents more than 75% of the inpatient beds in Spain, does not seem to seriously compromise the generalisation of the results. Yet, we identified that the mortality rate in our sample (6.2%) was higher than the one reported by the National Institute of Statistics (4.4%). This difference is probably explained by the lower participation in the PPS of hospitals with less severe patients such as psychiatric, long-term care, small or private hospitals. For this reason, and to obtain an accurate estimate, the PAF was computed using the National Institute of Statistics’ mortality rate. Finally, in our assessment, we did not consider HAIs that appeared after the day of the survey during the hospital stay nor those acquired in long-term care facilities. Concerning the outcome, readmissions, and deaths more than 30 days after the survey were also not taken into account. These exclusions could have led to misclassification and underestimation of the impact of HAIs on mortality. Although our study was limited to 30-day mortality, it is important to recognise that the consequences of HAIs often extend far beyond this period. Previous research has shown that patients who experience HAIs are at increased risk of hospital readmission or long-term morbidity, in addition to functional impairment, reduced quality of life and chronic health complications [3].

## Conclusion

Comprising over 100,000 inpatients, this study is, to our knowledge, the largest to assess attributable mortality for all types of HAIs. It emphasises the substantial impact of HAIs on patient mortality in Spain. Understanding the disease burden and the associated mortality risk of HAIs is crucial to highlight the consequences of HAIs and to guide the allocation of resources for strengthening infection prevention and control programmes nationwide. Given the 6, 774 annual deaths attributable to HAIs in Spain, urgent action is required to reinforce the implementation infection prevention programmes across all healthcare settings and to strengthen these programmes.

## Data Availability

All data collected within the Spanish point prevalence survey – EPINE (“Estudio de Prevalencia de Infecciones Nosocomiales en España”), including the data used in this study, are owned by the Spanish Society of Preventive Medicine, Public Health and Health Management. The datasets analysed are available from the corresponding author upon reasonable request and with permission from the data owner.

## Supplementary Data

332000 SupplementaryMaterial
