# Supplementary material for: Attributable mortality to healthcare-associated infections: a comprehensive nationwide assessment in Spain, 2022 and 2023
Source: Euro Surveill. 2026 Feb 19;31(7):2500139. doi: 10.2807/1560-7917.ES.2026.31.7.2500139 (PMC12923997; doi:10.2807/1560-7917.ES.2026.31.7.2500139)
Supplement: SupplementaryMaterial [file 25-00139_Supplementary_material.pdf]

This supplementary material is hosted by Eurosurveillance as supporting information alongside the article ‘Attributable mortality to healthcare-associated infections: a comprehensive nationwide assessment in Spain, 2022 and 2023’, on behalf of the authors, who remain responsible for the accuracy and appropriateness of the content. The same standards for ethics, copyright, attributions and permissions as for the article apply. Supplements are not edited by *Eurosurveillance* and the journal is not responsible for the maintenance of any links or email addresses provided therein.

Supplementary Table S1. Comparison of methods used to estimate the impact of HAIs across countries, 2001–2023

Country	Year	Study type	Population	Prevalence HAIs (%)	Mortality HAIs (%)	Mortality without HAIs (%)	Method for national estimates	National deaths due to HAIs
France [1]	2001–2003	Prospective cohort	7,086	NA	22.0	8.5	PAF: 1.7-3.0	NA
Norway [2]	2004–2011	Cross sectional	19,468	8.5	10.8	4.1	HR: 1.5	NA
Finland [3]	2005	Prevalence	8,234	8.5	9.8	NA	Incidence 6.4%<br>AFe: 31.9%	4,494
Belgium [4]	2007	Matched cohort	2,680	6.2	12.8	10.8	ARD: 2.8	3,477
Germany [5]	2011–2012	Prevalence	NA	NA	NA	NA	BCoDE	16,245
Greece [6]	2012	Prevalence-based cohort	8,247	7.0	9.7	2.8	Incidence 5.2%	NA
New Zealand [7]	2021	Prevalence	5,468	6.3	NA	NA	Model-based estimates<br>Incidence: 4.74	699
Spain (current study)	2022–2023	Prevalence-based cohort	107,781	7.8	11.0	5.7	AFe:41.2%<br>PAF: 3.2%	6,774

AFe: attributable fraction among the exposed; ARD: absolute risk difference; BCoDE: burden of communicable diseases in Europe methodology [8]; HAIs: healthcare-associated infections; HR: hazard ratio; NA: not available; PAF: population attributable fraction.

References

1. Fabbro-Peray P, Sotto A, Defez C, Cazaban M, Molinari L, Pinède M, et al. Mortality attributable to nosocomial infection: a cohort of patients with and without nosocomial infection in a French university hospital. Infect Control Hosp Epidemiol. 2007;28(3):265-72. http://dx.doi.org/10.1086/512626 PMID:17326016

2. Koch AM, Nilsen RM, Eriksen HM, Cox RJ, Harthug S. Mortality related to hospital-associated infections in a tertiary hospital; repeated cross-sectional studies between 2004-2011. Antimicrob Resist Infect Control. 2015;4(1):57. http://dx.doi.org/10.1186/s13756-015-0097-9 PMID:26719795

3. Kanerva M, Ollgren J, Virtanen MJ, Lyytikäinen O; Prevalence Survey Study Group. Estimating the annual burden of health care-associated infections in Finnish adult acute care hospitals. Am J Infect Control. 2009;37(3):227-30. http://dx.doi.org/10.1016/j.ajic.2008.07.004 PMID:19111367

4. Vrijens F, Hulstaert F, Devriese S, van de Sande S. Hospital-acquired infections in Belgian acute-care hospitals: an estimation of their global impact on mortality, length of stay and healthcare costs. Epidemiol Infect. 2012;140(1):126-36. http://dx.doi.org/10.1017/S0950268811000100 PMID:21320376

5. Zacher B, Haller S, Willrich N, Walter J, Abu Sin M, Cassini A, et al. Application of a new methodology and R package reveals a high burden of healthcare-associated infections (HAI) in Germany compared to the average in the European Union/European Economic Area, 2011 to 2012. Euro Surveill. 2019;24(46):1900135. http://dx.doi.org/10.2807/1560-7917.ES.2019.24.46.1900135 PMID:31771703

6. Kritsotakis EI, Kontopidou F, Astrinaki E, Roumbelaki M, Ioannidou E, Gikas A. Prevalence, incidence burden, and clinical impact of healthcare-associated infections and antimicrobial resistance: a national prevalent cohort study in acute care hospitals in Greece. Infect Drug Resist. 2017;10:317-28. http://dx.doi.org/10.2147/IDR.S147459 PMID:29066921

7. Morris AJ, Hensen M, Graves N, Cai Y, Wolkewitz M, Roberts SA, et al. The burden of healthcare-associated infections in New Zealand public hospitals 2021. Infect Control Hosp Epidemiol. 2024;45(10):1176-1182. http://dx.doi.org/10.1017/ice.2024.95 PMID:39363597

8. Colzani E, Cassini A, Lewandowski D, Mangen MJ, Plass D, McDonald SA, et al. A Software Tool for Estimation of Burden of Infectious Diseases in Europe Using Incidence-Based Disability Adjusted Life Years. PLoS One. 2017;12(1):e0170662. https://doi.org/10.1371/journal.pone.0170662 PMID: 28107447
